# Multimodal Transformer Model Using Time-Series Data to Classify Winter Road Surface Conditions

**DOI:** 10.3390/s24113440

**Published:** 2024-05-27

**Authors:** Yuya Moroto, Keisuke Maeda, Ren Togo, Takahiro Ogawa, Miki Haseyama

**Affiliations:** 1 Graduate School of Information Science and Technology, Hokkaido University, N-14, W-9, Kita-ku, Sapporo 060-0814, Japan; moroto@lmd.ist.hokudai.ac.jp; 2 Data-Driven Interdisciplinary Research Emergence Department, Hokkaido University, N-13, W-10, Kita-ku, Sapporo 060-0813, Japan; maeda@lmd.ist.hokudai.ac.jp; 3 Faculty of Information Science and Technology, Hokkaido University, N-14, W-9, Kita-ku, Sapporo 060-0814, Japan; togo@lmd.ist.hokudai.ac.jp (R.T.); ogawa@lmd.ist.hokudai.ac.jp (T.O.)

**Keywords:** deep learning, transformer, multimodal analysis, time-series processing, winter road surface condition

## Abstract

This paper proposes a multimodal Transformer model that uses time-series data to detect and predict winter road surface conditions. For detecting or predicting road surface conditions, the previous approach focuses on the cooperative use of multiple modalities as inputs, e.g., images captured by fixed-point cameras (road surface images) and auxiliary data related to road surface conditions under simple modality integration. Although such an approach achieves performance improvement compared to the method using only images or auxiliary data, there is a demand for further consideration of the way to integrate heterogeneous modalities. The proposed method realizes a more effective modality integration using a cross-attention mechanism and time-series processing. Concretely, when integrating multiple modalities, feature compensation through mutual complementation between modalities is realized through a feature integration technique based on a cross-attention mechanism, and the representational ability of the integrated features is enhanced. In addition, by introducing time-series processing for the input data across several timesteps, it is possible to consider the temporal changes in the road surface conditions. Experiments are conducted for both detection and prediction tasks using data corresponding to the current winter condition and data corresponding to a few hours after the current winter condition, respectively. The experimental results verify the effectiveness of the proposed method for both tasks. In addition to the construction of the classification model for winter road surface conditions, we first attempt to visualize the classification results, especially the prediction results, through the image style transfer model as supplemental extended experiments on image generation at the end of the paper.

## 1. Introduction

In snow-covered and cold regions, which account for approximately 60% of the land area in Japan, numerous winter-related traffic accidents occur due to weather conditions, e.g., snowfall. Approximately 90% of these accidents are slip-related incidents associated with winter road surface conditions due to snow accumulation and ice formation [1]. In this context, road managers need to undertake snow and ice control operations, e.g., snow removal and the spreading of anti-freezing agents by detecting or predicting road surface conditions to prevent slip accidents [1,2].

Previous studies have investigated the detection or prediction of winter road surface conditions [3,4,5,6,7,8]. In the literature [3], the road surface condition was predicted based on the heat balance theory using digital geographical data, which represent the shape of the land, including roads on computers; however, this method requires the analysis of digital geographical data related to the road, and it is difficult to collect and accumulate such data for all roads. In another study [7], the automatic detection of winter road surface conditions was realized using deep learning models trained on images captured by vehicle-mounted cameras. Similarly, winter road surface conditions were classified using hierarchical deep learning models applied to images also captured by vehicle-mounted cameras [8]. Here, to use images captured by vehicle-mounted cameras, it is necessary to drive on the road to be analyzed with vehicles equipped with cameras. To reduce such efforts, in the literature [4], data obtained from sensors and fixed-point cameras installed along roads were adopted to detect or predict the winter road surface conditions using rule-based methods. In addition, a previous study [5] achieved detection by classifying road surface conditions using differential methods based on images captured by fixed-point cameras installed along the road (hereafter referred to as road surface images). However, due to the temporal variability of road surfaces and roadside features, methods based on differential approaches require manual updating of the reference images. Thus, there is a demand for models that can classify road surface conditions automatically and accurately to facilitate precise detection and prediction. Several studies have focused on the winter road surface condition classification using the images captured by vehicle-mounted cameras [9,10,11]. The purpose of these studies is to help with the construction of autonomous vehicles; however, our purpose is to assist road managers in reducing winter-related traffic accidents using fixed-point cameras.

The multimodal analysis, which uses several information sources, e.g., images and natural languages, has attracted significant attention for improving the representational ability of models [12,13,14,15]. For example, contrastive language image pre-training has been proposed as the pre-training framework for the multimodal analysis of vision and language [16]. Another example is to use the texts obtained from Twitter in addition to images for image sentiment analysis [17]. In this way, most works on multimodal analysis have used vision and language modalities; however, in the classification task of winter road surface conditions, the text information does not exist, and the other information is needed for multimodal analysis. Then, we previously proposed an automated classification method for road surface conditions using a multimodal multilayer perceptron (MLP) using images and auxiliary data [18]. Concretely, in that study, the features calculated from multiple modalities, including road surface images and auxiliary data related to the road surface conditions such as temperatures and traffic volume, were concatenated and input to the MLP to classify the road surface conditions. The cooperative use of multiple modalities allows for mutual complementation between modalities, and we improved classification accuracy compared to using a single modality. However, in the previous study, we focused on the construction of machine learning models using multiple modalities and performed multimodal analysis through a simple feature concatenation process. As a result, this approach may have inherent limitations in terms of classification accuracy. Thus, further improvements in classification accuracy can be expected by introducing the following processes.

1. Time-series Analysis
  In the field of glaciology, a previous study [19] reported that snow accumulation extremes exhibit time-series variability. In addition, Hirai et al. [20] suggested that changes in road surface conditions are related to the transitions of these conditions over the past several timesteps. Thus, rather than relying on data from a single timestep (as in our previous study), using time-series data to classify road surface conditions is expected to improve the detection and prediction accuracy.
2. Feature Integration using Attention Mechanisms
  In our previous study, feature integration was performed by concatenating the features derived separately from image and auxiliary data and then inputting them into an MLP. On the other hand, in the machine learning field, Transformers [21,22,23,24], which are the novel machine learning architecture focusing on the relationship of input data, have attracted significant attention for the remarkable performance based on the strong representational ability. With the advancement of such Transformers, recent research on feature integration has demonstrated that intermediate fusion, which combines features in the intermediate layers of neural networks using cross-attention, achieves higher accuracy than traditional feature integration methods [25,26,27,28,29]. Cross-attention is an attention mechanism [21] with several inputs, which facilitates the compensation of heterogeneous features calculated from multiple modalities. As a result, the cross-attention module enhances the representational ability after integration, and the use of feature integration based on cross-attention is expected to further improve classification accuracy.

In this paper, we propose a new method for classifying winter road surface conditions using a multimodal transformer (MMTransformer) capable of processing time-series data. In the proposed method, image and auxiliary features are extracted from data spanning multiple timesteps, and feature integration considering temporal changes is performed by applying cross-attention. With cross-attention, correlations are calculated feature-wise for input data across multiple timesteps, and attention is computed for each timestep. This procedure enables feature integration that accounts for temporal changes in road surface conditions. Finally, the classification of winter road surface conditions is realized using an MLP. By exploring methods for integrating multiple modalities and introducing time-series processing, we aim to achieve improvements in accuracy in the detection and prediction of road surface conditions.

In addition, the proposed method can learn the relationship between the input data and the corresponding teacher labels, which are the labels related to winter road surface conditions for training the model. By altering the teacher labels assigned to the input data during training, the proposed method can be adapted to both detection and prediction tasks. In experiments conducted on real-world data, we evaluated the effectiveness of the proposed method for both detection and prediction tasks with two sets of teacher labels. One experiment was conducted with the teacher labels being the road surface condition corresponding to the input data, and the subsequent experiment was conducted with the teacher labels being the road surface condition a few hours after the input data. This dual approach allows for a comprehensive assessment of the capabilities of the proposed method in detecting the current road surface conditions and predicting future road surface conditions.

In addition to the experiments on the classification of winter road surface conditions, we conducted supplemental extended experiments on image generation to visualize the classification results, particularly the prediction results in the Appendix A. To help road managers make decisions, it can be effective to incorporate classification results and road surface images that visualize the results. In this study, we generated such images using an image style transfer model conditioned by road surface conditions. Through these supplemental experiments and visualizing the transferred images, we confirmed the potential of the image transfer model for road surface images.

The primary contributions of this study are summarized as follows.

1. A multimodal transformer model based on time-series processing and attention mechanisms is constructed to classify road surface conditions.
2. Experiments conducted to evaluate the road surface condition detection and prediction tasks verify the effectiveness of the proposed classification model.
3. The results of the supplemental extended experiments in the Appendix A demonstrate the potential of the image transfer model for road surface images.

The remainder of this paper is organized as follows. Section 2 introduces the data used in this study. The proposed method for the classification of winter road surface conditions is explained in Section 3. Then, the experimental results are reported in Section 4, and the supplemental extended experiments are discussed in Appendix A. Finally, Section 5 concludes the paper.

## 2. Data

In the following, we describe the data used in this study. We utilized road surface images acquired using fixed-point cameras and auxiliary data related to the road surface conditions. Specifically, these data were provided by the East Nippon Expressway Company Limited and were acquired from 2017 to 2019. The road surface images were captured at 20-min intervals from 1 December at 00:00 to 31 March at 23:40 each year. In addition, each road surface image was labeled with one of the following seven categories related to road surface conditions.

- Dry
  The road surface is free of snow, ice, and wetness.
- Wet
  The road surface is wet due to moisture.
- Black sherbet
  Tire tread marks are present, the snow contains a high amount of moisture, and the color of the road surface is black.
- White sherbet
  Tire tread marks are present, the snow contains a high amount of moisture, and the color of the road surface is white.
- Snow
  Snow has accumulated on the road surface, and the snow does not contain a high amount of moisture.
- Compacted snow
  There is no black shine and no tire tread marks.
- Ice
  Snow and ice are present on the road surface, and it appears black and shiny.

**Figure 1:**
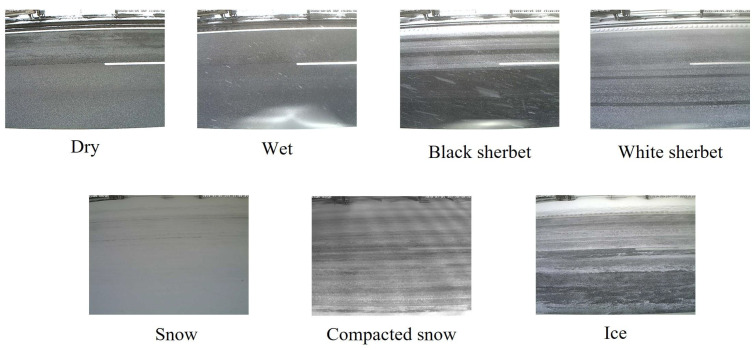
Road surface images for each winter road surface condition.

**Figure 2:**
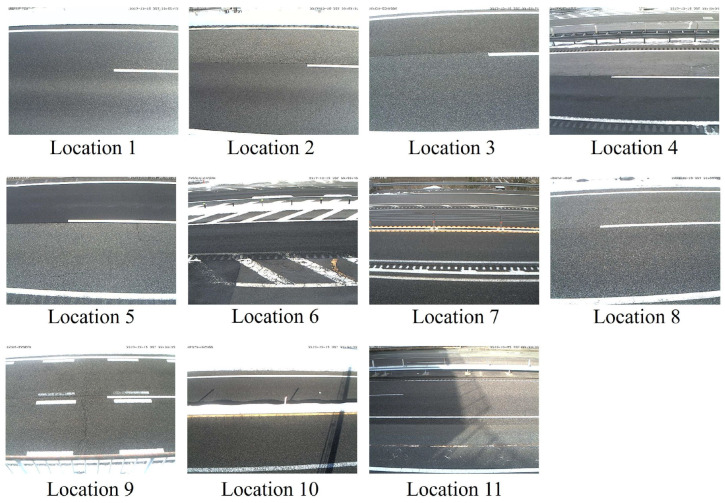
Locations where the road surface images were captured.

These labels were assigned by three experienced road managers, and they divided the annotation task and assigned the labels through visual inspections. Example road surface images for each category are shown in Figure 1, and the locations where the road surface images were captured are shown in Figure 2. Here, the image size is 640×480 pixels. Please note that road surface images, including vehicles, were considered for analysis because the vehicles did not cover the entire road surface in the images.

Table 1 shows the contents of the auxiliary data and the corresponding data types. As shown in Table 1, the “location of road surface images” and “weather forecast” are discrete information, while other data contents are represented as continuous values. As shown in Figure 1 and Table 1, the images and auxiliary data differ significantly; thus, a feature integration mechanism is required to complement the deficiencies in each modality. Thus, we attempt to improve the classification accuracy of road surface conditions by integrating multiple modalities at several timesteps.

## 3. Classification of Winter Road Surface Conditions Using MMTransformer

**Figure 3:**
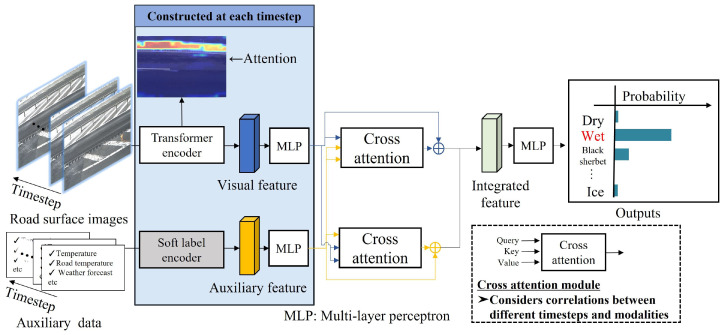
Overview of the proposed method.

**Figure 4:**
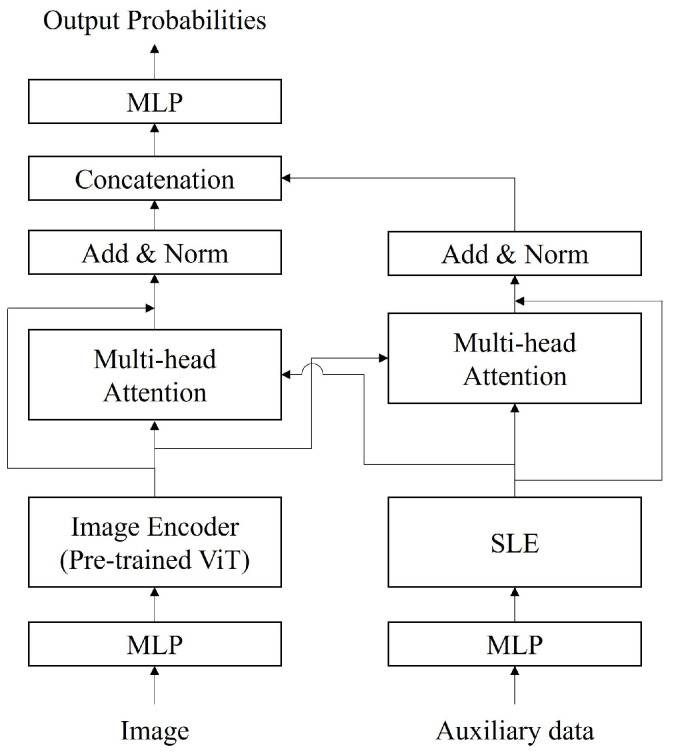
Flowchart of MMTransformer.

In this section, we describe the proposed method to classify winter road surface conditions based on the MMTransformer, which can process time-series data using images and auxiliary data at multiple timesteps as inputs. First, we construct encoders for both the image and auxiliary data at each timestep to extract relevant features. We then calculate the integrated features with the characteristics of both the image and auxiliary data by performing feature integration based on cross-attention. Finally, by inputting the integrated features into an MLP, we can classify the winter road surface conditions. An overview and flowchart of the proposed method are shown in Figure 3 and Figure 4, respectively. Please note that the proposed model is trained in an end-to-end manner, which allows the image encoder to be fine-tuned and the parameters in the MLP to be optimized simultaneously. In the following, we explain the methods for feature extraction and feature integration based on cross-attention in Section 3.1 and Section 3.2, respectively.

### 3.1. Feature Extraction

Here, we describe the method employed to construct the encoders used to extract the features from the image and auxiliary data.

#### 3.1.1. Visual Features

The proposed method utilizes output values from the intermediate layers of a pretrained deep learning model as visual features. For the deep learning model, we employ the Vision Transformer (ViT) [24] or its derivative methods [22,23], which have achieved high classification accuracy in image classification tasks. Training a model based on the ViT requires a large amount of training data; thus, we fine-tune a model pretrained on ImageNet [30] to extract the visual features with high representational ability from the road surface images.

**Figure 5:**
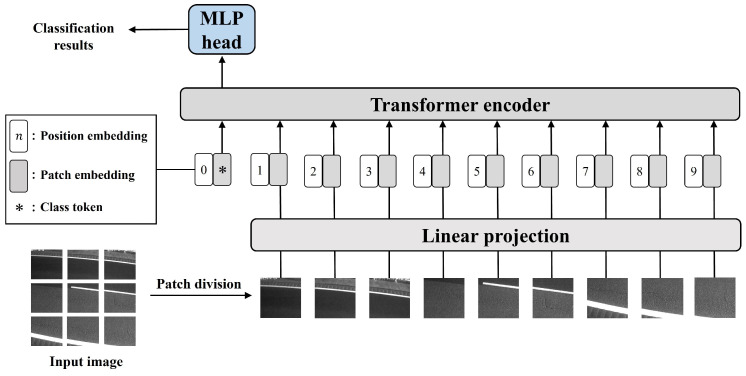
Architecture of the ViT [24].

In the ViT, as shown in Figure 5, patches obtained by dividing the images and position embeddings are input sequentially to linear layers and the Transformer encoder. The output values are calculated by the MLP head after the Transformer encoder. During fine-tuning of the ViT, transfer learning is performed on the Transformer encoder by replacing the MLP head. Specifically, in the proposed method, the visual feature $x_{t}^{\left(\mathrm{vis}\right)}\in R^{d_{\mathrm{vis}}}$ for image $V_{t}$ at timestep *t* (t=1,2,…,T, where *T* is the number of timesteps) is calculated as follows:

(1) $$\begin{array}{c} x_{t}^{\left(\mathrm{vis}\right)}=f\left(E_{\mathrm{vis}}\left(V_{t}\right)\right), \end{array}$$

where $E_{\mathrm{vis}}\left(\cdot \right)$ is the pretrained Transformer encoder in the ViT-based model, and f(·) is the MLP that calculates the visual features for input into the cross-attention mechanism. Thus, by employing an MLP head suitable for feature integration, it is possible to fine-tune the ViT-based model and train the cross-attention mechanism simultaneously.

#### 3.1.2. Auxiliary Features

**Figure 6:**
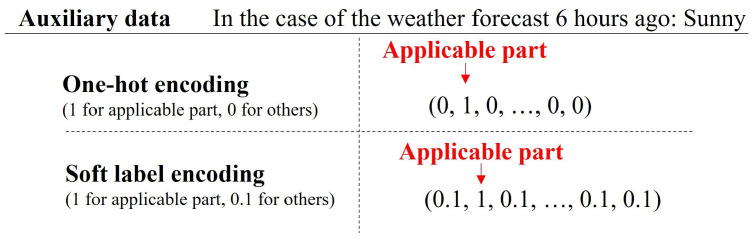
Example of SLE.

In the proposed method, the auxiliary data include both continuous quantitative variables, e.g., temperature and road temperature, and discrete qualitative variables, using nominal scales, e.g., location and weather conditions. Generally, in machine learning involving qualitative variables as inputs, one-hot encoding is used as a preprocessing method [31,32,33]. In one-hot encoding, elements equal to the number of items in the nominal scale are prepared, and the corresponding element is set to 1 (while others are set to 0). This procedure enables machine learning models to process qualitative variables. However, when one-hot encoded features ${\left\{x_{i}\right\}}_{i=0}^{n}$ are input to a neural network–based model, in the first layer of the forward propagation process, only the weights corresponding to the input elements with 1 are updated as follows:

(2) $$\begin{array}{c} a_{01}=\sum_{i=0}^{n}x_{i}W_{i0}+b_{0}, \end{array}$$

where ${\left\{W_{i0}\right\}}_{i=0}^{n}$ represents the weights corresponding to $x_{i}$, and $a_{01}$ is the output value at the 0th neuron in the first layer. As a result, the other weights corresponding to input elements with 0 are not updated, which makes it difficult to learn the correlations between the input elements. It has been reported that applying soft label encoding (SLE) to nominal scales in auxiliary data improves accuracy [33]. In SLE, the correlation between features can be learned by replacing the elements that are 0 in one-hot encoding with 0.1. Actually, in the literature [33], SLE (Figure 6) enabled the learning of correlations within auxiliary data and enhanced the representational ability. Thus, for the auxiliary data used in this study, applying SLE to the discrete qualitative variables is expected to improve the classification accuracy. Consequently, in the proposed method, SLE is applied to the discrete values, and a vector combined with continuous values is input to the MLP to calculate the auxiliary feature $x_{t}^{\left(\mathrm{aux}\right)}\in R^{d_{\mathrm{aux}}}$ at timestep *t*.

### 3.2. Feature Integration Based on Cross-Attention Mechanism

This section explains the cross-attention-based feature integration method. In the cross-attention module, the importance of each element in the features is determined using the query $q\in R^{T\times d_{m}^{\prime }}$, key $k\in R^{T\times d_{m}^{\prime }}$ and the value $v\in R^{T\times d_{m}^{\prime }}$ (m∈{vis,aux}, $d_{m}^{\prime }=d_{m}/h$). Here, *h* is a hyperparameter. The tuple (q, k, v) for each feature is calculated as follows:

(3) $$\begin{array}{cc} q^{m} & =X^{m}{W^{\left(q,m\right)}}^{⊤}, \end{array}$$

(4) $$\begin{array}{cc} k^{m} & =X^{m}{W^{\left(k,m\right)}}^{⊤}, \end{array}$$

(5) $$\begin{array}{cc} v^{m} & =X^{m}{W^{\left(v,m\right)}}^{⊤}, \end{array}$$

(6) $$\begin{array}{cc} \; & s.t.\;\;m\in \{\mathrm{vis},\mathrm{aux}\}, \end{array}$$

where $W^{\left(q,m\right)}\in R^{d_{m}^{\prime }\times d_{m}}$, $W^{\left(k,m\right)}\in R^{d_{m}^{\prime }\times d_{m}}$ and $W^{\left(v,m\right)}\in R^{d_{m}^{\prime }\times d_{m}}$ are the trainable parameters. In addition, $X^{m}=\left[{x_{1}^{m}}^{⊤},{x_{2}^{m}}^{⊤},\ldots ,{x_{T}^{m}}^{⊤}\right]\in R^{T\times d_{m}}$. Next, using the tuple (q, k, v) among the heterogeneous features, the cross-attention CA(·,·,·) is calculated as follows:

(7) $$\begin{array}{cc} \mathrm{CA}\left({q^{m}}^{\prime },k^{m},v^{m}\right)= & \left[{\mathrm{head}}_{1}^{\left(m^{\prime },m\right)},{\mathrm{head}}_{2}^{\left(m^{\prime },m\right)},\ldots ,{\mathrm{head}}_{h}^{\left(m^{\prime },m\right)}\right]W^{\left(o,m\right)}, \end{array}$$

(8) $$\begin{array}{cc} {\mathrm{head}}_{i}^{\left(m^{\prime },m\right)}= & \mathrm{Softmax}\left(\frac{{q^{m}}^{\prime }{k^{m}}^{⊤}}{\sqrt{d_{y}^{m}}}\right), \end{array}$$

(9) $$\begin{array}{cc} s.t.\;\;m^{\prime } & \neq m,\;i=1,2,\ldots ,h, \end{array}$$

where $W^{\left(o,m\right)}\in R^{hd_{m}^{\prime }\times d_{m^{\prime }}}$ is the trainable parameter. Finally, feature integration is performed by applying residual connections to each feature and the output values of the cross-attention mechanism as follows:

(10) $$\begin{array}{cc} {\hat{X}}^{m}= & X^{m}+\mathrm{CA}\left(q^{m},k^{m^{\prime }},v^{m^{\prime }}\right), \end{array}$$

(11) $$\begin{array}{cc} {\hat{X}}^{\mathrm{int}}= & [{\hat{X}}^{\mathrm{vis}},{\hat{X}}^{\mathrm{aux}}]. \end{array}$$

In the proposed method, vectorization is performed by applying mean pooling to the integrated feature ${\hat{X}}^{\mathrm{int}}$, which is then input to the MLP to output the final classification results. Thus, using cross-attention-based feature integration, the proposed method corrects features using heterogeneous data and processes time-series data across multiple timesteps. As a result, the proposed method improves the detection and prediction accuracy of winter road surface conditions.

## 4. Experiments

Experiments were conducted to verify the effectiveness of the proposed classification method based on MMTransformer. In the following, Section 4.1 describes the experimental dataset, Section 4.2 explains the experimental settings, and Section 4.3 presents the experimental results and a corresponding discussion.

### 4.1. Experimental Dataset

Here, we describe the dataset used in the experiments. The experiments utilized the winter road surface images and auxiliary data discussed in Section 4.1 to verify the effectiveness of the proposed method on real-world data. In addition, the seven categories (dry, wet, black sherbet, white sherbet, snow, compacted snow, and ice) were reorganized into three new categories, i.e., dry/wet, sherbet, and snow/compacted snow/ice, to detect and predict the winter road surface conditions from a practical perspective. The experiments were designed to confirm the effectiveness of using data across multiple timesteps to detect and predict winter road surface conditions. The classifications of road surface conditions were made for {0, 1, 3} hours later when inputting data at T(={1,3,5}) timesteps. Here, the data at one timestep were acquired at 20-min intervals. Please note that the input data were used on a per-timestep basis, and the teacher labels were used on an hourly basis. The number of samples for each road surface condition and the experimental settings are shown in Table 2, Table 3 and Table 4. In the multi-timestep experimental settings, missing data were imputed using the average values from the data at other timesteps. In addition, data from 2017 and 2018 were used as the training data without distinction of the location, and data from 2019 were used as the test data. Also, note that the number of samples in each category varied significantly in the training data; thus, to suppress the reduction in classification accuracy due to the imbalanced number of samples, random extraction was performed such that the number of samples belonging to each category was approximately equal. As a result, the number of samples in the training data was smaller than that of test data through such an undersampling operation.

### 4.2. Experimental Settings

Here, we describe the experimental settings. The MLP used in the proposed method comprised three layers, and the feature dimensions of the images and auxiliary data were set to $d_{\mathrm{vis}}=16$ and $d_{\mathrm{aux}}=16$, respectively. For the Transformer encoder in the proposed method, we employed the ViT-B/16 model [24], which was pretrained on ImageNet [30]. For the loss function, cross-entropy loss was used, and for the optimization method, the Adam optimizer [34] with a learning rate of 0.001 was employed. During the training, the batch size was set to 8, and the number of epochs was set to 10. Moreover, we set h=4 as the hyperparameter.

To verify the effectiveness of the cross-attention–based feature integration implemented in the proposed method, we compared a method (Concatenation) that does not employ cross-attention by replacing Equation (11) with the following expression:

(12) $$\begin{array}{cc} {\hat{X}}^{\mathrm{int}} & =[X^{\mathrm{vis}},X^{\mathrm{aux}}]. \end{array}$$

To evaluate the performance of the detection and prediction results, accuracy, macro precision, macro recall, and macro F1 metrics were considered, which are frequently used in the machine learning field for multiclass classification tasks. Each evaluation metric is calculated as follows:

- Accuracy
  (13) $$\begin{array}{c} \mathrm{Accuracy}=\frac{\sum_{l=1}^{L}{\mathrm{TP}}_{l}}{\sum_{l=1}^{L}\left({\mathrm{TP}}_{l}+{\mathrm{FP}}_{l}\right)}. \end{array}$$
- Macro Precision
  (14) $$\begin{array}{cc} \mathrm{Macro}\mathrm{Precision} & =\frac{1}{L}\sum_{l=1}^{L}{\mathrm{Precision}}_{l}, \end{array}$$
  (15) $$\begin{array}{cc} {\mathrm{Precision}}_{l} & =\frac{{\mathrm{TP}}_{l}}{{\mathrm{TP}}_{l}+{\mathrm{FP}}_{l}}. \end{array}$$
- Macro Recall
  (16) $$\begin{array}{cc} \mathrm{Macro}\mathrm{Recall} & =\frac{1}{L}\sum_{l=1}^{L}{\mathrm{Recall}}_{l}, \end{array}$$
  (17) $$\begin{array}{cc} {\mathrm{Recall}}_{l} & =\frac{{\mathrm{TP}}_{l}}{{\mathrm{TP}}_{l}+{\mathrm{FN}}_{l}}. \end{array}$$
- Macro F1
  (18) $$\begin{array}{cc} \mathrm{Macro}F1 & =\frac{1}{L}\sum_{l=1}^{L}{F1}_{l}, \end{array}$$
  (19) $$\begin{array}{cc} {F1}_{l} & =\frac{2\times {\mathrm{Recall}}_{l}\times {\mathrm{Precision}}_{l}}{{\mathrm{Recall}}_{l}+{\mathrm{Precision}}_{l}}. \end{array}$$

Here, ${\mathrm{TP}}_{l}$ and ${\mathrm{FN}}_{l}$ represent the number of true positive samples and false negative samples for the *l*th category, respectively, and ${\mathrm{FP}}_{l}$ denotes the number of false positive samples for the *l*th category.

### 4.3. Results and Discussion

#### 4.3.1. Effectiveness of Time-Series Analysis

The experimental results obtained with different numbers of timesteps in the input data are shown in Table 5, Table 6 and Table 7. Under all experimental conditions, the increase in the number of timesteps resulted in a higher macro F1 score, and we confirmed the effectiveness of using multiple timesteps when detecting and predicting the winter road surface conditions. On the other hand, when comparing MMTransformer w/5 with MMTransformer w/3 in Table 5, the macro Precision score decreased. Similarly, when comparing MMTransformer w/5 with MMTransformer w/3 in Table 7, the macro Recall score decreased. These score decreases were caused by differences in ${\mathrm{FP}}_{l}$ for macro Precision and in ${\mathrm{FN}}_{l}$ for macro Recall; however, both ${\mathrm{FP}}_{l}$ and ${\mathrm{FN}}_{l}$ should be evaluated for the classification model. Thus, we mainly focused on the harmonic mean of macro Precision and macro Recall, i.e., macro F1, and discussed the difference in the performance based on the macro F1. Thus, the effectiveness of time-series analysis with input data at multiple timesteps in the proposed method has been verified.

#### 4.3.2. Effectiveness of Cross-Attention Mechanism

The experimental results comparing the proposed method with other methods are shown in Table 8, Table 9 and Table 10. As can be seen, the macro F1 score of the proposed method surpasses that of the compared methods, which confirms the effectiveness of the MMTransformer. Specifically, by comparing MMTransformer and Concatenation, we verified that the cross-attention-based feature integration is effective for the classification of winter road surface conditions. On the other hand, when comparing MMTransformer w/5 with Concatenation w/5 in Table 10, the macro Recall score decreased. As well as macro Precision in Table 5 and macro Recall in Table 7, we mainly focused on the harmonic mean of macro Precision and macro Recall, i.e., macro F1, and discussed the difference in the performance based on the macro F1.

**Figure 7:**
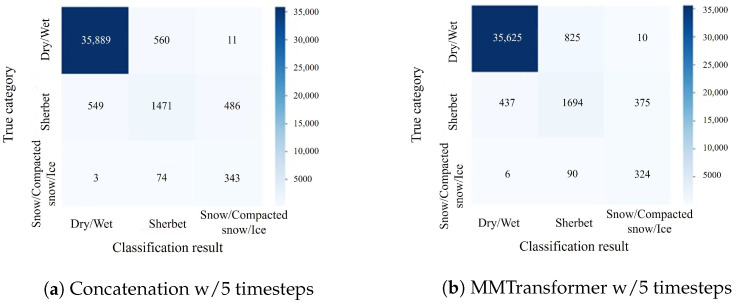
Confusionmatrix for the experiment to immediately predict (detect) the road surface condition (corresponding to Table 8).

**Figure 8:**
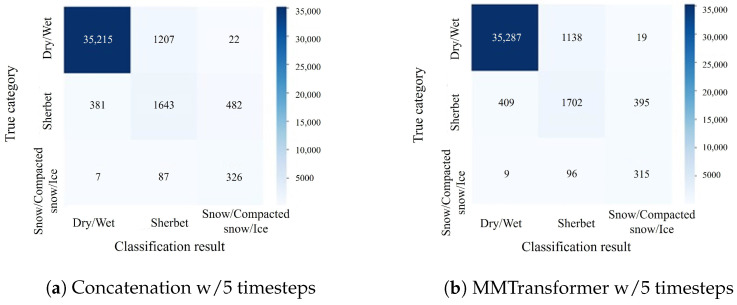
Confusion matrix for the experiment to predict the road surface condition one hour later (corresponding to Table 9).

**Figure 9:**
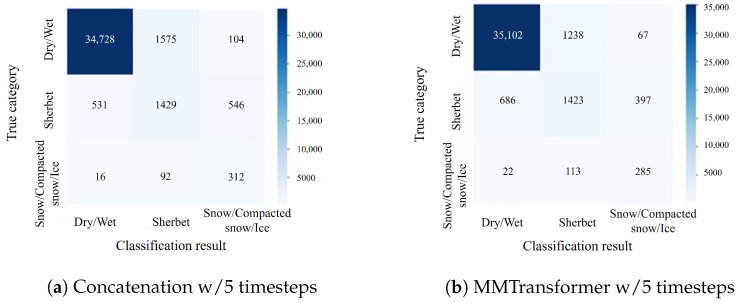
Confusionmatrix for the experiment to predict the road surface condition three hours later (corresponding to Table 10).

Thus, the effectiveness of using feature integration based on the cross-attention mechanism as the feature integration method has been verified. In addition, confusion matrices for the classification results of Concatenation w/5 timesteps and MMTransformer w/5 timesteps are shown in Figure 7, Figure 8 and Figure 9. In Figure 7 and Figure 8, the number of samples classified correctly for the dry/wet and snow/compacted snow/ice categories is approximately the same for both the MMTransformer and Concatenation. For the sherbet category, the MMTransformer outperformed the Concatenation considerably in terms of the number of correctly classified samples. In Figure 9, the number of correctly classified samples for the sherbet and snow/compacted snow/ice categories is similar; however, the MMTransformer outperformed the Concatenation considerably in the dry/wet category. These results confirm that the MMTransformer can predict winter road surface conditions more accurately than the Concatenation. However, when predicting the winter road surface conditions three hours later, as shown in Figure 9, there was no significant improvement in terms of classification accuracy for the important sherbet and snow/compacted snow/ice categories, which are critical for the effective detection and prediction of winter road surface conditions. Thus, improving the accuracy of predictions for winter road surface conditions at later times remains a challenge for future work.

#### 4.3.3. Qualitative Evaluation through Visualization

In the MMTransformer, the output values obtained from the ViT model’s intermediate layers are used as image features. The ViT model employs an attention mechanism that recognizes important regions in images automatically and applies weighting to these regions. To achieve this, attention rollout [35], which presents the regions focused on by ViT through visualizing the weights in the attention mechanism, has been proposed. The regions presented by attention rollout are expected to serve as a basis for the rationale behind the classification results obtained by the ViT. In the proposed method, by observing the regions for winter road surface images, it is possible to gain insights into the relationship between the winter road surface images and winter road surface conditions and to use this information to enhance the performance of the classification model.

**Figure 10:**
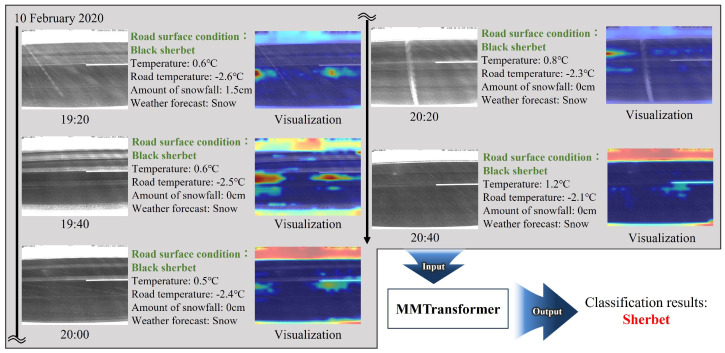
Example visualizationobtained by applying attention rollout to the ViT model, i.e., the image encoder in MMTransformer.

Figure 10 shows a visualization example obtained by applying attention rollout to the ViT encoder in MMTransformer, where redder regions are of higher interest in MMTransformer, and bluer regions are of lower interest. Here, the visualization was performed for MMTransformer w/5 timesteps in the experimental setting to detect the winter road surface conditions. As can be seen, there is more attention on the snow at the roadside at 20:00 and 20:40, and there is consistent attention to certain parts of the road surface over all timesteps. These observations imply that MMTransformer w/5 timesteps recognizes the presence of snow on the roadside but correctly identifies the road surface condition as sherbet due to the lesser amount of snow compared to the snow/compacted snow/ice conditions. From this result, it can be inferred that MMTransformer w/5 timesteps performs detection and prediction by focusing on the snow accumulation on the surface of the road in the images. Thus, by outputting the visualization results for the input images, we can gain insights into the relationship between the winter road surface images and the road surface conditions, and these insights can be used to enhance the performance of detection and prediction models.

## 5. Conclusions

This paper has proposed the MMTransformer method, which uses time-series data to detect and predict winter road surface conditions. The proposed method enhances the representational ability of the integrated features by performing feature correction through mutual complementation between modalities based on a cross-attention-based feature integration method for multiple modalities, e.g., road surface images and auxiliary data. In addition, by introducing time-series processing for the input data at multiple timesteps, the proposed method can integrate features in consideration of the temporal changes in winter road surface conditions. As a result, the proposed method improves the classification accuracy of winter road surface conditions by introducing a new integration for multiple modalities and time-series processing.

Experiments confirmed that the proposed MMTransformer method achieves high accuracy in classifying winter road surface conditions and is effective for both the detection and prediction tasks by varying the teacher labels. In addition, using attention rollout for visualization, we expected to provide additional insights into the relationship between road surface images and road surface conditions. In this way, as the experimental findings, it was implied that attention rollout works well for the multimodal classification model of winter road surface conditions. The visualization in the image encoder can be utilized to enhance the classification model when detecting and predicting road surface conditions, and the experimental findings discussed in this paper have demonstrated the potential of this technique.

On the other hand, confusion matrices indicate that performance improvement was slight for the data belonging to sherbet or snow/compacted snow/ice categories since the road surface images belonging to sherbet or snow/compacted snow/ice categories were visually similar to those of each other category. Such limitations caused by visual similarity can be solved by effectively leveraging non-visual information, including auxiliary data, which remains in future works.

## Figures and Tables

**Table 1:** Auxiliary data and corresponding data types.

Data Content	Data Type
Location of road surface images	Discrete
Temperature	Continuous
Road temperature	Continuous
Amount of snowfall	Continuous
Traffic volume	Continuous
Average of vehicle speed	Continuous
Weather forecast six hours ago	Discrete
Temperature forecast six hours ago	Continuous
Snowfall forecast six hours ago	Continuous
Weather forecast 12 h ago	Discrete
Temperature forecast 12 h ago	Continuous
Snowfall forecast 12 h ago	Continuous
Weather forecast 18 h ago	Discrete
Temperature forecast 18 h ago	Continuous
Snowfall forecast 18 h ago	Continuous
Weather forecast 24 h ago	Discrete
Temperature forecast 24 h ago	Continuous
Snowfall forecast 24 h ago	Continuous

**Table 2:** Breakdown of experimental data used to immediately predict (detect) the road surface condition (0 h later).

	Number of Timesteps Used as Input					
	1		3		5	
Road Surface Condition	Training	Test	Training	Test	Training	Test
Dry/Wet	6000	35,771	6000	36,388	6000	36,460
Sherbet	5829	2474	5921	2505	5939	2506
Snow/Compacted snow/Ice	4614	418	4740	420	4747	420
Sum	16,443	38,663	16,661	39,313	16,686	39,386

**Table 3:** Breakdown of experimental data used to predict the road surface condition one hour later.

	Number of Timesteps Used as Input					
	1		3		5	
Road Surface Condition	Training	Test	Training	Test	Training	Test
Dry/Wet	6000	35,738	6000	36,370	6000	36,444
Sherbet	5838	2477	5935	2504	5948	2506
Snow/Compacted snow/Ice	4604	416	4730	420	4739	420
Sum	16,442	38,631	16,665	39,294	16,687	39,370

**Table 4:** Breakdown of experimental data used to predict the road surface condition three hours later.

	Number of Timesteps Used as Input					
	1		3		5	
Road Surface Condition	Training	Test	Training	Test	Training	Test
Dry/Wet	6000	35,703	6000	36,332	6000	36,407
Sherbet	5836	2475	5942	2504	5954	2506
Snow/Compacted snow/Ice	4604	416	4730	420	4741	420
Sum	16,428	38,593	16,667	39,256	16,695	39,333

**Table 5:** Experimental results obtained when varying the number of timesteps in the experiment to immediately predict (detect) the road surface condition.

Method	Accuracy	Macro Precision	Macro Recall	Macro F1
MMTransformer w/1 timestep	0.954	0.689	0.768	0.702
MMTransformer w/3 timesteps	0.958	0.710	0.791	0.735
MMTransformer w/5 timesteps	0.956	0.698	0.808	0.740

**Table 6:** Experimental results obtained when varying the number of timesteps in the experiment to predict the road surface condition one hour later.

Method	Accuracy	Macro Precision	Macro Recall	Macro F1
MMTransformer w/1 timestep	0.941	0.633	0.774	0.678
MMTransformer w/3 timesteps	0.944	0.636	0.791	0.683
MMTransformer w/5 timesteps	0.948	0.667	0.799	0.717

**Table 7:** Experimental results obtained when varying the number of timesteps in the experiment to predict the road surface condition three hours later.

Method	Accuracy	Macro Precision	Macro Recall	Macro F1
MMTransformer w/1 timestep	0.919	0.560	0.746	0.612
MMTransformer w/3 timesteps	0.926	0.579	0.747	0.627
MMTransformer w/5 timesteps	0.936	0.625	0.737	0.666

**Table 8:** Comparison of results in experiments to immediately predict (detect) road surface conditions.

Method	Accuracy	Macro Precision	Macro Recall	Macro F1
Concatenation w/5 timesteps	0.957	0.697	0.796	0.722
MMTransformer w/5 timesteps	0.956	0.698	0.808	0.740

**Table 9:** Comparison of results in experiments to predict road surface conditions one hour later.

Method	Accuracy	Macro Precision	Macro Recall	Macro F1
Concatenation w/5 timesteps	0.944	0.647	0.799	0.701
MMTransformer w/5 timesteps	0.948	0.667	0.799	0.717

**Table 10:** Comparison of results in experiments to predict road surface conditions three hours later.

Method	Accuracy	Macro Precision	Macro Recall	Macro F1
Concatenation w/5 timesteps	0.927	0.590	0.756	0.644
MMTransformer w/5 timesteps	0.936	0.625	0.737	0.666

**Figure A1:**
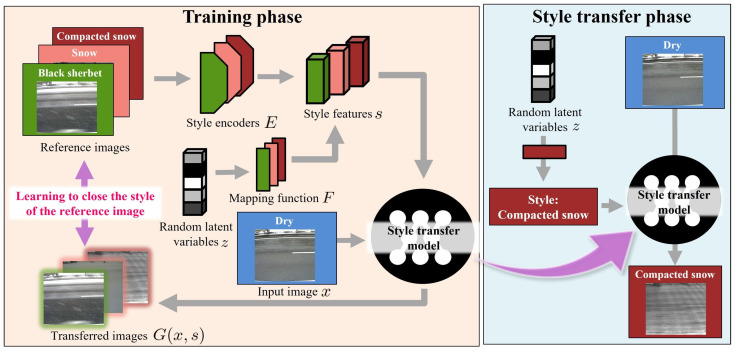
Overview of image generation using the style transfer model. It should be noted that the discriminator *D* is used to close the styles of the reference images and those of the transferred images.

**Figure A2:**
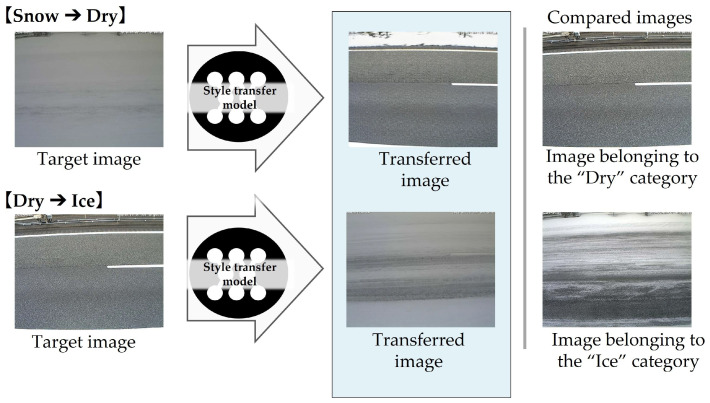
Examples of road surface condition transformation in road surface images using StarGAN v2 model. For reference and comparison, a road surface image with the same label as the transferred image is also shown.

## Data Availability

Experimental data cannot be disclosed.

## Appendix A. Supplemental Extended Experiments on Image Generation

### Appendix A.1. Background

In this section, to visualize the classification results, especially in terms of the prediction results, we conducted supplemental extended experiments focusing on image generation.

In the classification of road surface conditions, we assume that the workflow of the classification model presents only the classification results of road surface conditions to road managers. Such a workflow makes it difficult to visualize the detailed state of the road surface. Thus, by visually presenting the road surface conditions a few hours later in addition to the classification results, it is expected that more informed decisions will be made with the knowledge and experience of road managers. In this way, the visualization of classification results facilitates effective decision support for snow and ice removal operations.

In the computer vision field, tasks involving the style transformation of images have traditionally been addressed [36,37]. Such style transfer tasks involve learning the relationships between domains to transform a target image into a desired image style. For example, by learning the relationship between a domain of images capturing a horse and a domain of images capturing a zebra, the image style transfer model can output an image where the patterns on the body of the horse are transformed into that of the zebra. Similarly, for road surface images, it is possible to transfer an input image to an image with the style of the predicted road surface condition using the image style transfer model. As a result, the image generation reflecting the style of the predicted road surface conditions can be realized using image style transfer with input road surface images. The generated images hold promise in terms of providing visual decision support for road managers making snow and ice removal decisions.

In the supplemental extended experiments, we first attempted to generate images using the style of specific road surface conditions using the image style transfer model. Specifically, since there are multiple categories of road surface conditions, we performed multidomain style transfer for each category as a domain. Here, we used StarGAN v2 [38] as the style transfer model. The StarGAN v2 model is a well-known multidomain style transfer model that achieves efficient multidomain style transfer by training a single generator to handle multiple domains to acquire domain-specific features.

### Appendix A.2. Image Style Transfer

In this subsection, we summarize the method used to transform the road surface conditions in the road surface images using the StarGAN v2 model. An overview of the image style transfer process using the StarGAN v2 model is shown in Figure A1. When the input image and domain are denoted x∈X and y∈Y, respectively, StarGAN v2 attempts to transform the input image *x* into the style of each domain *y* using a single generator *G*. Here, X and Y represent the set of images and the set of domains after transformation, respectively. To generate images that reflect the style of each domain from a single generator, domain-specific style features are input along with the input image, and the StarGAN v2 model controls the style of the image output by the generator *G*. In the following, we explain the modules used in the StarGAN v2 model, i.e., the generator, mapping function, style encoder, discriminator, and the objective function for optimization.

In the StarGAN v2 model, the generator *G* transforms the input image *x* into image G(x,s) using style features *s* obtained from either the mapping function *F* or the style encoder *E*. By incorporating adaptive instance normalization [39,40] into the generator, StarGAN v2 enables style transfer using the style features *s*. As a result, by calculating the style code *s* to represent domain-specific features, it is possible to generate images that reflect the style of multiple domains using only a single generator (without the need to construct separate generators for each domain).

The mapping function *F* calculates the style features *s* from the random latent variables *z*. Specifically, by utilizing an MLP with multiple output branches corresponding to each road surface condition, the style features are calculated as s=F(z). This multitask architecture enables efficient calculation of the style features.

The style encoder *E* extracts the style features *s* from the image *x* as s=E(x). Using the style features calculated by inputting a reference image into the style encoder, it is possible to transform the input image into an image that reflects the style of the reference image.

The discriminator *D* distinguishes between images that belong to the target domain and images that are transformed by the generator when an image is input. Here, efficient learning is achieved by adopting a multitasking architecture similar to the mapping function *F* and style encoder *E*.

In the StarGAN v2 model, to enable a single generator to output images corresponding to the styles of multiple domains, the entire model is trained by optimizing the following objective function:

(A1) $$\begin{array}{c} \min_{G,F,E}\max_{D}L_{adv}+\lambda_{sty}L_{sty}-\lambda_{ds}L_{ds}+\lambda_{cyc}L_{cyc}, \end{array}$$

where $\lambda_{sty}$, $\lambda_{ds}$, and $\lambda_{cyc}$ are hyperparameters, and $L_{adv}$ is the adversarial loss used to acquire domain-specific style features and enhance the quality of the generated images. In addition, $L_{sty}$ is the style reconstruction loss, which is used to enable the extraction of style features that correspond to each domain from images. This reconstruction loss is inspired by the literature [41,42]; however, the main difference lies in the ability to extract style features for multiple domains using a single style encoder. $L_{ds}$ is the diversity regularization loss [43,44] used to ensure the diversity of the generated images, and $L_{cyc}$ is the cycle consistency loss [45,46,47], which is used to preserve domain-invariant features in the input image in the transformed image. Using these different losses, it is possible to generate images that correspond to the styles of multiple domains using only a single generator.

### Appendix A.3. Experimental Results

We conducted the supplemental extended experiment using the StarGAN v2 model to transform the surface conditions in the road surface images using a style transfer model. The road surface conditions targeted in this experiment and the number of training images labeled for each condition are shown in Table A1. Please note that the number of road surface conditions differed from that described in Section 4.1 to confirm that the image style transfer models can represent diverse road surface conditions. Here, the purpose of this section is to confirm the potential of applying image style transfer models to road surface images, and we experimentally used as many road surface conditions as possible. In addition, the labels were assigned not by the classification models, e.g., MMTransformer, but by experienced road managers (Section 2) to evaluate the image style transfer model without misclassification effects.

**Table A1:** Number of training images labeled with each road surface condition.

Road Surface Condition	Dry	Wet	Black Sherbet	White Sherbet	Snow	Ice	Compacted Snow	Sum
	39,807	45,778	13,568	2910	2313	320	2045	106,741

In this experiment, the hyperparameters $\lambda_{sty}$, $\lambda_{ds}$, and $\lambda_{cyc}$ were all set to 1, and the dimensionality of the random latent variables was set to 16. In addition, the dimensionality of the style features was set to 64. The model was optimized using the Adam optimizer [34] with 100,000 epochs and a batch size of 8. The learning rates for *D*, *E*, and *G* were set to 0.0001, and the learning rate for *F* was set to 0.000001.

Figure A2 shows examples of road surface condition transfer in road surface images and the corresponding compared images. Here, the compared images are road surface images labeled with the same conditions as the transferred images. The experimental results confirm that the transferred images visually resemble the compared images. In addition, the ability to acquire visually distinct images accurately supports the potential to generate road surface images with transferred road surface conditions by training a style transfer model on road surface images.

### Appendix A.4. Conclusions

In addition to the construction of the classification model for winter road surface conditions, we conducted supplemental extended experiments on image generation to visualize the classification results, especially the prediction results. The experimental results demonstrate that the generated images reflect the specified styles. Thus, the classification results can be represented as images using image style transfer models to help road managers make decisions. However, comparative experiments and quantitative evaluations were not conducted in this study, although we have supported the potential of using an image style transfer model for road surface images. Thus, the construction of an image style transfer model specific to road surface images and its evaluation remains an issue for future work.
